# Analysis of HBV basal core promoter/precore gene variability in patients with HBV drug resistance and HIV co-infection in Northwest Ethiopia

**DOI:** 10.1371/journal.pone.0191970

**Published:** 2018-02-06

**Authors:** Yeshambel Belyhun, Uwe Gerd Liebert, Melanie Maier

**Affiliations:** 1 Institute of Virology, Medical Faculty, Leipzig University, Leipzig, Germany; 2 School of Biomedical and Laboratory Sciences, College of Medicine and Health Sciences, University of Gondar, Gondar, Ethiopia; University of Cincinnati College of Medicine, UNITED STATES

## Abstract

**Background:**

We recently reported complex hepatitis B virus (HBV) drug resistant and concomitant vaccine escape hepatitis B surface antigen (HBsAg) variants during human immunodeficiency virus (HIV) co-infection and antiretroviral therapy (ART) exposure in Ethiopia. As a continuation of this report using the HBV positive sera from the same study participants, the current study further analyzed the HBV basal core promoter (BCP)/precore (PC) genes variability in patients with HBV drug resistance (at tyrosine-methionine-aspartate-aspartate (YMDD) reverse transcriptase (RT) motifs) and HIV co-infection in comparison with HBV mono-infected counterparts with no HBV drug resistant gene variants.

**Materials and methods:**

A total of 143 participants of HBV-HIV co-infected (n = 48), HBV mono-infected blood donors (n = 43) and chronic liver disease (CLD) patients (n = 52) were included in the study. The BCP/PC genome regions responsible for HBeAg expression from the EcoRI site (nucleotides 1653–1959) were sequenced and analyzed for the BCP/PC mutant variants.

**Results:**

Among the major mutant variants detected, double BCP mutations (A1762T/G1764A) (25.9%), Kozak sequences mutations (nt1809-1812) (51.7%) and the classical PC mutations such as A1814C/C1816T (15.4%), G1896A (25.2%) and G1862T (44.8%) were predominant mutant variants. The prevalence of the double BCP mutations was significantly lower in HIV co-infected patients (8.3%) compared with HBV mono-infected blood donors (32.6%) and CLD patients (36.5%). However, the Kozak sequences BCP mutations and the majority of PC mutations showed no significant differences among the study groups. Moreover, except for the overall BCP/PC mutant variants, co-prevalence rates of each major BCP/PC mutations and YMDDRT motif associated lamivudine (3TC)/entecavir (ETV) resistance mutations showed no significant differences when compared with the rates of BCP/PC mutations without YMDD RT motif drug resistance gene mutations. Unlike HIV co-infected group, no similar comparison made among HBV mono-infected blood donors and CLD patients since none of them developed the YMDD RT motif associated 3TC/ETV resistance mutations. However, HBV mono-infected blood donors and CLD patients who had no any drug resistance gene variants developed comparable G1862T (60.6% vs. 65.1%) and G1896A (24.2% vs. 11.6%) PC gene mutations.

**Conclusion:**

No correlation observed between the BCP/PC genome variability and the YMDD RT motif associated HBV drug resistance gene variants during HIV co-infection. Nevertheless, irrespective of HIV co-infection status, the higher records of the BCP/PC gene variability in this study setting indicate a high risk of potential HBeAg negative chronic HBV infection in Northwest Ethiopia.

## Background

During chronic hepatitis B (CHB) infection, hepatitis B e antigen (HBeAg) is used as a marker of active viral proliferation that might lead to active liver damage [1, 2]. The loss of HBeAg and development of antibodies to HBeAg (anti-HBe) are usually associated with low HBV DNA levels. In other words, it is the end of viral replication and clinical remission of liver disease [3] and considered as a favorable sign for patients with CHB [2]. However, after seroconversion, a stage of HBV infection characterized by persistently elevated HBV DNA levels, active histological and biochemical activity, and active liver disease called HBeAg negative CHB infection could appear and can lead to cirrhosis and hepatocellular carcinoma (HCC) [4]. Nowadays, the HBeAg-negative CHB represents an increasing clinical challenge. Its prevalence has increased over the last few decades and became a common type of HBV infection in many parts of the world [5].

The BCP and PC gene mutations were reported to cause HBeAg-negative CHB infection by affecting HBeAg expression which ultimately leads to HBeAg loss and negativity with a severe form of chronic liver disease progression [6–9]. Moreover, the presences of such genes change often need long-term HBV therapy that makes patients susceptible to nucleos(t)ide analogues drug resistance gene evolution [10]. In the opposite, few reports [11–13] showed that HBV therapy using 3TC in particular resulted in rapid development of the BCP mutants in HBeAg-positive patients, but that leads to reversion of BCP mutants to wild-type in HBeAg-negative patients [14,15]. On the other hand, *in vitro* studies [16, 17] showed that co-presence of BCP and PC mutant variants substantially enhanced the replication fitness of drug-resistant HBV mutants particularly for 3TC, ETV and ADV [16, 18–20]. These could be taken as an advantage for the virus fitness since the polymerase gene mutations in the YMDD RT motifs (mainly associated with 3TC resistance) were reported to reduce viral replication [15, 16]. The synergetic effects of YMDD RT motif drug resistance and BCP/PC genes variability in keeping the viral replication fitness, however, have usually been reported [16, 18, 19] under the influence of no HIV co-infection and ART exposure. In this regard, although HIV co-infection was reported to alter the natural history of HBV infection by increasing viral replication, mutant evolution and risk of liver disease progression [21, 22], little is known about HBV BCP/PC genomic heterogeneity during HIV co-infection. Recently, we reported a high prevalence and complex HBV drug resistance pattern with concomitant heterogeneous vaccine escape HBsAg variants as the result of HIV co-infection and continuous ART exposure in Ethiopia [23]. As a continuation of the above report using the HBV positive sera from the same study subjects, the current study determined the correlation between the BCP/PC genes variability and YMDD RT motifs associated HBV drug resistance gene variants during HIV co-infection in comparison with HBV mono-infected counterparts with no any drug-resistant gene mutant variants.

## Materials and methods

### Study participants

A total of 143 study participants was included in the current study with a representation of three groups, 33.6% (48) HBV-HIV co-infected, 30.1% (43) HBV mono-infected blood donors and 36.4% (52) HBV mono-infected CLD patients who attended University of Gondar Teaching Hospital, Debretabor Hospital and Gondar Poly Health Centre in northwest Ethiopia. The HIV-HBV co-infected groups were from known HIV carriers recruited from HIV/AIDS Clinics during their ART follow up time from the above health institutions. Patients clinically defined as acute and chronic liver disease attendants were included in the CLD study group. The blood donors were not clinically characterized except for taking their past medical histories such as blood transfusion, liver disease illness and sexually transmitted infections. The study population enrollment, demographic data and sera collection, and screening in each respective study group were reported before [23]. In addition, the study participants were characterized before by complex HBV drug resistance patterns conferring resistance to 3TC, ETV and ADV and concomitant vaccine invasive HBsAg mutant variants [23]. In particular, the HBV-HIV co-infected group was characterized by the YMDD RT associated 3TC/ETV drug resistance gene mutations (rtM204V/I) which appeared as multiple combinations with other mutant variants, such as rtV173L and rtL180M [23]. Moreover, this group included ART naïve and ART exposed study participants in which the latter group showed a higher rate of the YMDD RT motif drug resistance gene variants [23].

### Serological and virological characteristics

Demographic and clinical data as well as blood sera were collected after the study was approved by the Institutional Ethical Review Board of University of Gondar in Ethiopia (Ref. No: RCS/V/P/05/372/2013). Informed consent from each study participant and permission from the University of Gondar teaching Hospital, Debretabor Hospital, and Gondar Poly Health Center (Ref. No: CMHS/08/30/2013) were also obtained before data collection. Details of patients/clients’ serum collection, HBV and HIV screening were well described before [23]. The sera were tested for HBeAg and antibody (HBeAb) using commercially available enzyme immunoassay Architect System kits (Abbott Diagnostics, Wiesbaden, Germany).

### HBV nucleic acid extraction and quantification

HBV DNA was extracted using 600μl sera from HBsAg positive study subjects by applying Abbott mSample Preparation System DNA on the m2000sp system (Abbott Molecular, Des Plaines, IL, USA). The HBV DNA viral load was quantified using a Real Time HBV assay on the Abbott m2000rt system (Abbott Molecular, Des Plaines, IL, USA).

### PCR amplification and direct sequencing of BCP/PC genome region

With a brief modification of PCR condition and reaction mixture, the BCP/PC genome regions from the EcoRI site (nucleotide (nt) 1653–1959) were amplified using a set of primers which were used before [24]. The BCP/PC nucleotide codons covered the partial X protein, full precore and partial core protein (C) genome regions. In brief, the viral DNA was amplified using Promega Taq DNA polymerase (Promega, Madison, WI, USA) by sense (5’-GCATGGAGACCACCGTGAAC-3’) (1606–1625 from EcoRI site) and anti-sense (5’-GGAAAGAAGTCCGAGGGCAA-3’) (1974–1955 from EcoRI site)) primers in the first round PCR and subsequently by the inner primers; sense 5’-CATAAGAGGACTCTTGGACT-3’ (1653–1672 from EcoRI site) and antisense 5’-GGCAAAAAACAGAGTAACTC-3’ (1959–1940 from EcoRI site). The PCR thermal cycling was done with parameters of an initial denaturation at 94°C for 30 sec, followed by 30 cycles of 30 sec at 94°C denaturation, 30 sec at 55°C annealing, and 60 sec at 72°C extension followed by a final extension of 2 min at 72°C. The PCR reaction mixture concentration and conditions were similar for both the first and second-round PCR with an exception of increasing the cycles to 35 in the latter round. Finally the amplicons were visualized with 1.5% agarose gel electrophoresis and purified using Wizard SV Gel and PCR Clean-Up System (Promega, Mannheim, Germany) and directly sequenced in both directions using second round primers and ABI Prism Big Dye Terminator cycle sequencing reaction kit and ABI Prism 3500 Genetic Analyzer (Applied Biosystems, Foster City, CA, USA).

### BCP/PC gene variability analysis

The nucleotide sequences obtained in this study were manually edited and aligned using Geneious software version 6.2.1 (http://www.geneious.com). GenBank sequences showing the highest matching scores with the current study sequences in the National Center for Biotechnology Information (NCBI) Basic Local Alignment Search Tool (BLAST) were retrieved and retained for both separate and multiple sequence alignment used for both genotyping and mutational analysis. Basically, genotyping was performed using three methods. First, each of the query sequence was compared against all aligned GenBank HBV reference sequences of each genotype. Secondly, genotype analysis was performed using the genotyping tool available from the NCBI website (http://www.ncbi.nlm.nih.gov/projects/genotyping/formpage.cgi) and finally confirmed using phylogenetic tree analysis (S1 Fig). The phylogenetic tree was constructed in MEGA6 (http://www.megasoftware.net/) using the Neighbor-Joining method.

Similarly, during gene mutation analysis, each study sequences were compared against separate and multiple aligned references sequences to assess the variation at the nucleotide positions of interest. For sequence alignments, the EditSeq and Megalign programs from the DNAstar software package (Lasergene Inc., Madison, WI, USA) were used. Moreover, for further determination and confirmation, the mutation analysis was repeated by aligning and comparing with the known wild-type GenBank reference sequences (Genotype A: X70185, Genotype D: X72702 and Genotype E: X75657) using MULTALIN software version 5.4.1 as described before by Chauhan et al [2]. During sequences analysis, much emphasis was given for the detection of mutations affecting HBeAg expression at the BCP transcriptional (BCP double mutations; A1762T/G1764A) and translational genes (Kozak sequences mutants, nt1809-1812) and PC initiation (1814–1816), translational stop codon (G1896A with C1858T) and post translational mutant gene (G1862T) [8]. In addition to the above major BCP/PC mutational genome sites, nucleotide changes to the rest of BCP functional genome regions were also examined with special focus on to TA rich genome regions TA1 (nt 1750–1755), TA2 (nt 1758–1762), TA3 (nt1771-1775), TA4 (nt 1788–1795) and initiation sites of PC mRNA at nt 1788–1791 and pregenomic RNA at nt 1817–1821 [2]. The presence of the nt ‘‘C” at position nt1858 was also checked for genotype A and nt ‘‘T” for genotype D [2]. Finally, the mutant variants detected were compared among the study groups with special emphasis on HIV co-infected patients who were described before as having HBV drug resistance gene mutations at their YMDD RT motif due to rtM204V/I in particular and the rest RT motif of the polymerase gene in general [23]. The BCP/PC nucleotide sequences analyzed in the current study are available in the GenBank/EMBL/DDBJ data bases with accession numbers KY463528-KY463670.

### Statistical analysis

The categorical data and median (interquartile range, IQR) of continuous variables were compared by Chi-square and Mann-Whitney tests, respectively using GraphPad Prism (version version 5.01, 2007). A p-value of less than 0.05 was considered as statistically significant.

## Results

### Demographic and clinical characteristics

The HIV co-infected group consisted of patients with different clinical WHO stages (Table 1) and almost half of them (47.9%) were in their ART follow-up for a median (IQR) of 3.8 (1.7–7.5) years. The CLD study group included patients with a manifestation of ascites, cirrhosis, HCC and/or liver complications from other infections. In addition, patients with acute hepatitis liver illness were part of this group (Table 1).

**Table 1:** Demographic, virological and clinical characteristics of study subjects.

Variables	HIV co-infected (n = 48)	HBV mono-infected (n = 95)		Total (n = 143)	P -value*
		Liver disease patients (n = 52)	Blood donors (n = 43)		
**Demographic characteristics**	**n(%)**	**n(%)**	**n(%)**	**n(%)**	
Age: Median(Interquartile range, IQR)	34(28–41)	35(25–45)	28(22–35)	32(25–40)	**0.004**
Sex: Male	22(45.8)	40(76.9)	37(86.0)	99(69.2)	**<0.001**
Female	26(54.2)	12(23.1)	6(14.0)	44(30.8)	
**Virological characteristics**					
HBeAg status: Negative	26(54.2)	31(59.6)	32(74.4)	89(62.2)	0.12
Positive	22(45.8)	21(40.4)	11(25.6)	54(37.8)	
Plasma HBV DNA viral load (logIU/ml):Median(IQR)	6.7(3.1–8.4)	5.5(4.1–6.8)	3.3(2.8–3.9)	4.4(3.2–7.2)	**0.05**
HBV genotypes: A	26(54.2)	31(59.6)	29(67.4)	86(60.1)	0.67
D	21(43.8)	21(40.4)	14(32.6)	56(39.2)	
E	1(2.1)	0(0.0)	0(0.0)	1(0.7)	
HBV drug resistance mutations^†^:Undetected	20(41.7)	43(82.7)	33(76.7)	96(67.1)	**<0.001**
Detected	28(58.3)	9(17.3)	10(23.3)	47(32.9)	
HBsAg escape mutations^†^: Undetected	8(16.7)	15(28.8)	13(30.2)	36(25.2)	0.25
Detected	40(83.3)	37(71.2)	30(69.8)	107(74.8)	
**ART related clinical characteristics** **(for HIV co-infected patients only)**					
WHO staging: I & II	40(83.3)	-	-	40(83.3)	-
III & IV	8(16.6)	-	-	8(16.6)	-
ART: Experienced	23(47.9)	-	-	23(47.9)	-
Naïve	25(52.1)	-	-	25(52.1)	-
ART follow up (in years) Median(IQR)	3.8(1.7–7.5)	-	-	3.8(1.7–7.5)	-
**Clinical status (for liver disease patients only)**					
Undefined	-	17(32.7)	-	17(32.7)	-
Hepatocelluar carcinoma	-	8(15.4)	-	8(15.4)	-
Ascites	-	6 (11.5)	-	6 (11.5)	-
Cirrhosis	-	4(7.7)	-	4(7.7)	-
Acute hepatitis +Jaundice+ chronic hepatitis	-	8(15.4)	-	8(15.4)	-
Complications with other infections/conditions^&^	-	9(17.3)	-	9(17.3)	-

^†^Major mutations conferring multi-drug resistance and HBsAg escape mutations as reported before [24].

^&^Schistosomiasis and hepato/splenomegaly conditions.

Column cells indicated with a sign ‘–’ means not applicable.

*P-value indicated the statistical variances among the three groups.

### Virological characteristics

**Fig 1:**
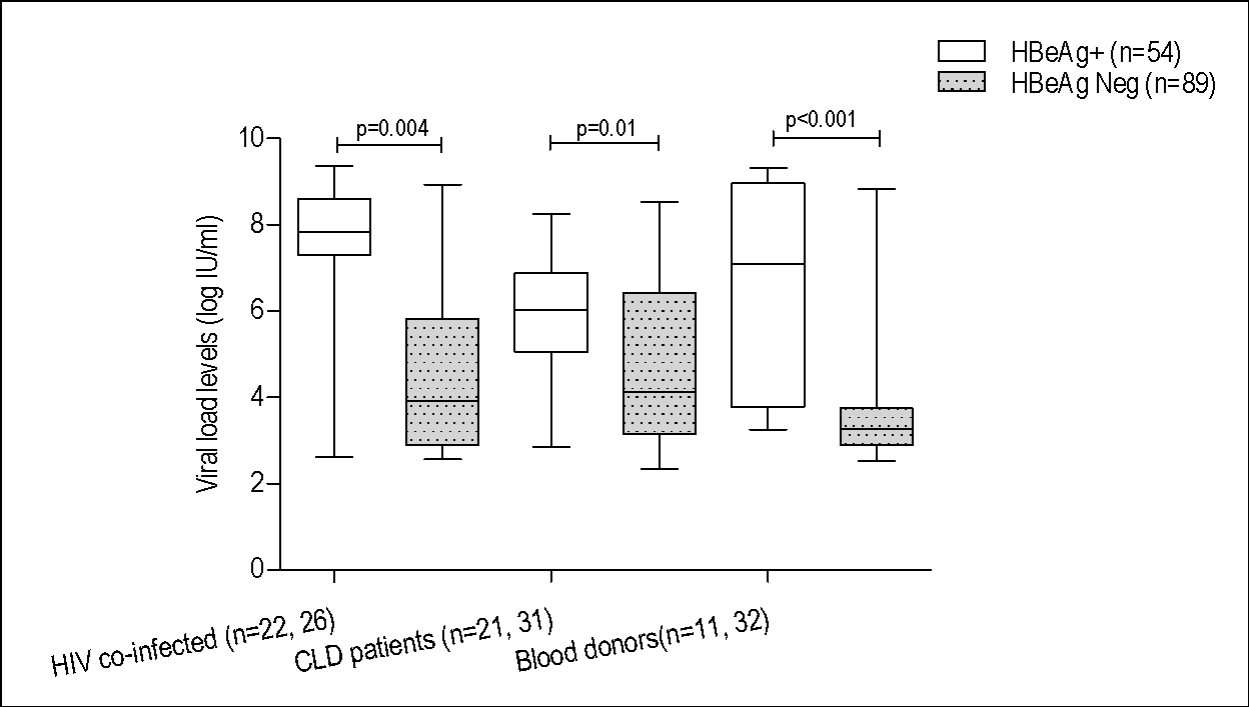
Comparison of HBV viral load levels between HBeAg status.

The overall HBeAg negativity rate was 62.2% (89) and showed no significant difference among the three study groups (Table 1). The overall median (IQR) HBV viral load level of the 143 study subjects was 4.4 (3.2–9.4) log IU/ml which was significantly higher in HBeAg positive subjects (7.1 (5.4–8.3) log IU/ml) than in negatives (3.7(3.0–4.7) log IU/ml) (Fig 1).

The genotypic analysis of the 143 sequences showed that HBV genotype A (60.1%) was predominant, followed by genotype D (39.2%) and genotype E (0.7%) (Table 1).

### Analysis of the BCP/PC genome variability

Overall, 79.7% (114) of the study subjects showed any of the BCP mutants detected (Table 2). The frequency of the major BCP mutants; A1762T and G1764A was 28.7% and 35.0%, respectively (Table 2).

**Table 2:** Frequency distribution of BCP and PC mutations among study groups, HBeAg status and HBV genotypes.

	Mutations	Study groups, n(%)				P-value	HBeAg status, n (%)			P-value	Genotypes, n (%)				
		HIV co-infected (n = 48)	Blood donors (n = 43)	CLD patients (n = 52)	Total (n = 143)		Positive (n = 54)	Negative (n = 89)	Total (n = 143)		A(n = 86)	D(n = 56)	E(n = 1)	Total	P-value*
**BCP mutations**	C1674T	22(45.8)	13(30.2)	17(32.7)	52(36.4)	0.24	14(25.9)	38(42.7)	52(36.4)	0.07	3(3.5)	48(85.7)	1(100)	52(36.4)	**<0.001**
	A1676T	18(37.5)	13(27.9)	17(32.7)	47(32.9)	0.62	12(22.2)	35(39.3)	47(32.9)	**0.05**	3(3.5)	43(76.8)	1(100)	47(32.9)	**<0.001**
	C1678T/A	19(39.6)	11(25.6)	17(32.7)	47(32.9)	0.37	12(22.2)	35(39.3)	47(32.9)	**0.05**	3(3.5)	44(78.6)	0(0.0)	47(32.9)	**<0.001**
	C1703A	20(41.7)	12(27.9)	17(32.7)	49(34.3)	0.37	15(27.8)	34(38.2)	49(34.3)	0.28	9(10.5)	39(69.6)	1(100)	49(34.3)	**<0.001**
	G1719T	23(47.9)	17(39.5)	18(34.6)	58(40.6)	0.40	15(27.8)	43(48.3)	58(40.6)	**0.03**	4(4.7)	53(94.6)	1(100)	58(40.6)	**<0.001**
	A1727G	6(12.5)	5(11.6)	1(1.9)	12(8.4)	0.11	8(9.0)	4(7.4)	12(8.4)	0.74	3(3.5)	9(16.1)	0(0.0)	12(8.4)	**0.03**
	C1730	1(2.1)	1(2.3)	1(1.9)	3(2.1)	0.99	2(3.7)	1(1.1)	3(2.1)	0.66	1(1.2)	2(3.6)	0(0.0)	3(2.1)	0.61
	T1741C	1(2.1)	0(0.0)	3(1.9)	4(2.8)	0.22	2(3.7)	2(2.2)	4(2.8)	0.63	2(2.4)	2(3.6)	0(0.0)	4(2.8)	0.87
	A1752C/G/T	6(12.5)	4(9.3)	2(3.8)	12(8.4)	0.29	2(3.7)	10(11.2)	12(8.4)	0.13	3(3.5)	9(16.1)	0(0.0)	12(8.4)	**0.03**
	T1753V*	7(14.6)	10(23.3)	10(19.2)	27(18.9)	0.57	3(5.6)	24(27)	27(18.9)	**<0.001**	7(8.1)	20(35.7)	0(0.0)	27(18.9)	**<0.001**
	G1757A	20(41.7)	11(25.6)	18(34.6)	49(34.3)	0.31	13(24.1)	36(40.4)	49(34.3)	0.07	3(3.5)	45(80.4)	1(100)	49(34.3)	**<0.001**
	T1758C	0(0.0)	0(0.0)	1(1.9)	1(0.7)	0.41	0(0.0)	1(1.1)	1(0.7)	0.99	0(0.0)	1(1.8)	0(0.0)	1(0.7)	0.46
	A1760C	0(0.0)	1(2.3)	0(0.0)	1(0.7)	0.31	0(0.0)	1(1.1)	1(0.7)	0.99	1(1.2)	0(0.0)	0(0.0)	1(0.7)	0.71
	A1761C	2(4.2)	1(2.3)	0(0.0)	3(2.1)	0.35	0(0.0)	3(3.4)	3(2.1)	0.29	1(1.2)	2(3.6)	0(0.0)	3(2.1)	0.61
	A1762T	4(8.3)	16(37.3)	21(40.4)	41(28.7)	**0.001**	15(27.8)	26(29.2)	41(28.7)	0.99	33(38.4)	8(14.3)	0(0.0)	41(28.7)	**0.01**
	G1763A	0(0.0)	1(2.3)	2(3.8)	3(2.1)	0.40	0(0.0)	3(3.4)	3(2.1)	0.29	1(1.2)	2(3.6)	0(0.0)	3(2.1)	0.61
	G1764A	6(12.5)	19(44.2)	25(48.1)	50(35.0)	**<0.001**	21(38.9)	29(32.6)	50(35.0)	0.56	38(44.2)	12(21.4)	0(0.0)	50(35.0)	**0.02**
	C1766T	5(10.4)	4(9.3)	12(23.1)	21(14.7)	0.10	10(18.5)	11(12.4)	21(14.7)	0.44	17(19.8)	4(7.1)	0(0.0)	21(14.7)	0.06
	T1767A	1(2.1)	1(2.3)	0(0.0)	2(1.4)	0.56	1(1.9)	1(1.1)	2(1.4)	0.99	2(2.4)	0(0.0)	0(0.0)	2(1.4)	0.50
	T1768A	3(6.2)	0(0.0)	10(19.2)	13(9.1)	**0.004**	7(13)	6(6.7)	13(9.1)	0.34	8(9.3)	5(8.9)	0(0.0)	13(9.1)	0.90
	T1771C	0(0.0)	0(0.0)	1(1.9)	1(0.7)	0.41	1(1.9)	0(0.0)	1(0.7)	0.99	0(0.0)	1(1.8)	0(0.0)	1(0.7)	0.46
	A1772C	1(0.7)	0(0.0)	0(0.0)	1(0.7)	0.37	0(0.0)	1(1.1)	1(0.7)	0.99	0(0.0)	1(1.8)	0(0.0)	1(0.7)	0.46
	T1773C	22(45.8)	14(32.6)	17(32.7)	53(37.1)	0.30	14(25.9)	39(43.8)	53(37.1)	**0.03**	3(3.5)	49(87.5)	1(100)	53(37.1)	**<0.001**
	G1775A	0(0.0)	1(2.3)	0(0.0)	1(0.7)	0.31	1(1.9)	0(0.0)	1(0.7)	0.38	0(0.0)	1(1.8)	0(0.0)	1(0.7)	0.46
	C1799T	0(0.0)	0(0.0)	1(1.9)	1(0.7)	0.41	0(0.0)	1(1.1)	1(0.7)	0.99	0(0.0)	1(1.8)	0(0.0)	1(0.7)	0.46
	T1809G	21(43.8)	10(23.3)	16(30.8)	47(32.9)	0.11	13(24.1)	34(38.2)	47(32.9)	0.12	2(2.3)	44(78.6)	1100)	47(32.9)	**<0.001**
	C1810A/T	0(0.0)	2(4.7)	2(3.8)	4(2.8)	0.34	2 (3.7)	2(2.2)	4(2.8)	0.63	4(4.7)	0(0.0)	0(0.0)	4(2.8)	0.25
	A1811C	4(8.3)	7(16.3)	3(5.8)	14(9.8)	0.21	2(3.7)	12(13.5)	14(9.8)	0.08	12(14.0)	2(3.6)	0(0.0)	14(9.8)	0.11
	T1812C	22(45.8)	12(27.9)	21(40.4)	55(38.5)	0.20	16(29.6)	39(43.8)	55(38.5)	0.13	6(7.0)	48(85.7)	1(100)	55(38.5)	**<0.001**
**PC mutations**	A1814C	7(14.6)	7(16.3)	8(15.4)	22(15.4)	0.98	3(5.6)	19(21.3)	22(15.4)	**0.02**	15(17.4)	7(12.5)	0(0.0)	22(15.4)	0.59
	C1816T	1(2.1)	1(2.3)	0(0.0)	2(1.4)	0.56	0(0.0)	2(2.2)	2(1.4)	0.53	0(0.0)	2(3.6)	0(0.0)	2(1.4)	0.21
	C1845G/T	0(0.0)	0(0.0)	1(1.9)	1(0.7)	0.41	1(1.9)	0(0.0)	1(0.7)	0.38	1(1.2)	0(0.0)	0(0.0)	1(0.7)	0.71
	A1850T	22(45.8)	9(20.9)	15(28.8)	46(32.2)	**0.03**	13(24.1)	33(37.1)	46(32.2)	0.15	10(11.6)	35(62.5)	1(100)	46(32.2)	**<0.001**
	C1858T	23(47.9)	14(32.6)	21(40.4)	58(40.6)	0.33	16(29.6)	42(47.2)	58(40.6)	0.06	9(10.5)	48(85.7)	1(100)	58(40.6)	**<0.001**
	G1862T	22(45.8)	21(48.8)	21(40.4)	64(44.8)	0.70	20(37.0)	44(49.4)	64(44.8)	0.20	29(32.9)	34(60.7)	1(100)	64(44.8)	**<0.001**
	G1888A/T	14(29.2)	12(27.9)	17(32.7)	43(30.1)	0.87	24(44.4)	19(21.3)	43(30.1)	**0.01**	39(45.3)	4(7.1)	0(0.0)	43(30.1)	**<0.001**
	G1896A	9(18.8)	14(32.6)	13(25)	36(25.2)	0.32	4(7.4)	32(36.0)	36(25.2)	**<0.001**	5(5.8)	31(55.4)	0(0.0)	36(25.2)	**<0.001**
	G1899A	9(18.8)	9(20.9)	10(19.2)	28(19.6)	0.96	6(11.1)	22(24.7)	28(19.6)	0.08	10(11.6)	18(32.1)	0(0.0)	28(19.6)	**0.004**

*V-stands for A or C or G.

**Fig 2:**
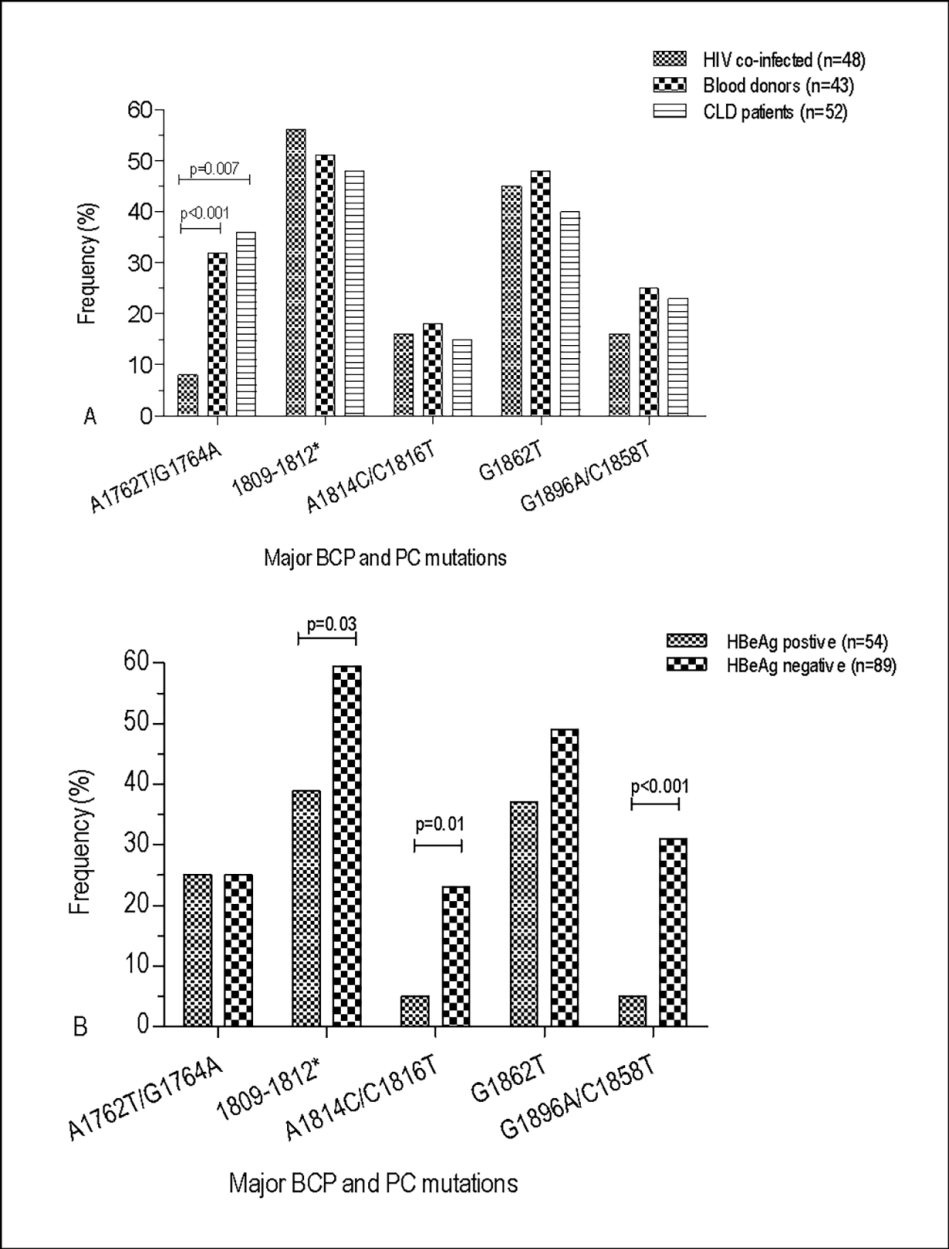
Prevalence comparison of the major BCP and PC mutations affecting HBeAg expression at the transcriptional (A1762T/G1764A), translational (nt 1809–1812), PC initiation (nt 1814–1816), stop codon G1896A (with C1858T) or post translational levels (G1862T) among study groups (Fig 2A) and HBeAg status (Fig 2B). *The translational or the Kozak sequence mutants include T1809G, C1810A/T, A1811C and T1812C.

The Kozak sequence mutants (nt 1809–1812) with a prevalence of 51.7% (74) includes T1809G (32.9%), C1810A/T (2.8%), A1811C (9.8%) and T1812C (38.5%) (Table 2). Many of the BCP mutations showed no significant difference among the study groups except for the A1762T, G1764A and T1768A mutant variants (Table 2). The double BCP mutations (A1762T/G1764A) accounted to 25.9% and showed no statistical difference between blood donors (32.6%) and CLD patients (36.5%), but were least detected in HIV co-infected patients (8.3%) (Fig 2A).

Similarly, the majority of the BCP mutations showed no significant difference among HBeAg status with the exception of A1676T (32.9%), C1678T/A (32.9%), G1719T (40.6%) and T1773C (18.9%) (Table 2) and the Kozak sequence mutant variants, which were higher in subjects with HBeAg negative status (Fig 2B). Unlike the major double BCP mutants (A1762T and G1764A), which were significantly higher among subjects with genotype A, many of the BCP mutant variants were either significantly higher in subjects with genotype D or showed no difference among genotypes (Table 2).

The classical PC mutant variants such as A1814C/C1816T (15.4%), G1862T (40.4%) and G1896/C1858T (23.1%) (Fig 2A) and many of other common PC mutants (Table 2) were detected with an overall frequency of 84.6% (121). The prevalence comparison of the major classical PC mutations (Fig 2A) and other PC mutations (Table 2) showed no significant difference. Only mutant variants A1814C/C1816T (23.5 vs. 5.6%) and G1896/C1858T (31.5 vs. 5.6%) were significantly higher in HBeAg negative cases than positives (Fig 2B). The majority of PC mutations were observed among study subjects with HBV genotype D (Table 2). Group and case-specific multiple BCP/PC mutations detections are presented in Table 3 and S1 Table.

**Table 3:** Comparison of HBV BCP/ PC nucleotide sequence changes with respect to HBV drug resistance associated polymerase gene mutations from HIV co-infected patients (n = 28), liver disease patients (n = 9) and blood donors (n = 10).

Study groups	Nucleotide positions						BCP transcriptional & translational genes																												PC initiation & post translational genes									
							1	1	1	1	1	1	1	1	1	1	1	1	1	1	1	1	1	1	1	1	1	1	1	1	1	1	1	1	1	1	1	1	1	1	1	1	1	1
							6	6	6	7	7	7	7	7	7	7	7	7	7	7	7	7	7	7	7	7	7	7	7	7	8	8	8	8	8	8	8	8	8	8	8	8	8	9
							7	7	7	0	1	2	3	4	5	5	5	5	6	6	6	6	6	6	6	7	7	7	7	9	0	1	1	1	1	1	4	5	5	6	8	9	9	3
	Isolates	Genotype	VL	HBeAg	HBsAg escape mutations*	Pol mutations*	4	6	8	3	9	7	0	1	2	3	7	8	0	1	2	3	4	6	8	1	2	3	5	9	9	0	1	2	4	6	5	0	8	2	8	6	9	4
Ref.strians	X70185	A					T	A	C	C	G	G	C	T	A	T	G	T	A	A	A	G	G	C	T	T	A	T		C	G	C	A	C	A	G	C	A	C	G	G	G	G	A
	X72702	D					T	.	.	A	T	.	.	.	.	.	A	.	.	.	.	.	.	.	.	.	.	C		.	.	.	.	.	.	.	.	T	T	.	.	.	.	.
	X75657	E					T	T	.	A	T	A	.	C	.	.	A	.	.	.	.	.	.	.	.	.	.	C		.	.	.	.	.	.	.	.	T	T	.	.	.	.	.
HBV-HIV coinfected	ETH1150	D	6.54	+	T127P, P120S, K160N	T128I, R153W,P237T	.	T	.	A	T	G	.	.	.	.	A	.	.	.	.	.	.	.	.	.	.	C		.	G	.	.	C	.	.	.	T	T	.	.	.	.	.
	ETH1480	A	2.72	-	T118A, Q129H, P127T	I233V	C	.	.	.	G	.	.	.	.	.	.	.	.	.	.	.	.	.	.	.	.	.		.	.	.	.	.	.	.	.	.	.	T	.	.	.	.
	ETH1490	D	2.61	-	T118A, Q129H, P127T	I233V	C	.	.	A	T	.	.	.	.	C	A	.	.	.	.	.	.	.	.	.	.	C		.	G	.	.	C	C	T	.	T	T	.	.	.	R	T
	ETH1640	D	2,66	-	T118A, Q129H, P127T	I233V	.	T	T	A	T	.	.	.	.	.	A	.	.	.	.	.	.	.	.	.	.	C		.	G	.	.	C	.	.	.	T	T	.	.	A	.	T
	ETH1650	D	9.02	+	T127L, E164V	L82S,P237T,Q267L	.	.	A	A	T	G	A	.	.	.	A	.	.	.	.	.	.	.	.	.	.	C		.	G	.	.	C	.	.	.	T	T	.	T	.	.	T
	**ETH1660**	**E**	**7.2**	**+**	**E164D,I195M**	**V173L, L180M, M204V**	.	**T**	.	**A**	**T**	.	.	**C**	.	.	**A**	.	.	.	.	.	.	.	.	.	.	**C**	** **	.	**G**	.	.	**C**	.	.	.	**T**	**T**	.	.	.	.	**T**
	ETH1670	D	8.89	+	T118A, Y134F, P127T	V191I	.	T	T	A	T	.	.	.	.	.	A	.	.	.	.	.	.	.	.	.	.	C		.	G	.	.	C	.	.	.	T	T	.	.	.	.	.
	ETH1680	D	2.63	+	T118A, Q129H, P127T	V84A, I233V	.	T	T	.	T	.	.	.	.	.	A	.	.	.	.	.	.	.	.	.	.	C		.	G	.	.	C	.	.	.	T	T	.	.	.	.	.
	ETH1860	D	7.97	-	T118A, P127T, E164G	L229M	.	T	T	A	T	.	.	.	C	.	A	.	.	.	.	.	.	.	.	.	.	C		.	G	.	.	C	.	.	.	T	T	.	.	A	A	T
	**ETH1870**	**A**	**4.6**	**-**	**Q129R, G130N,E164D**	**V173L, L180M, M204V**	**C**	.	.	**A**	.	.	.	.	.	.	.	.	.	.	.	.	.	.	.	.	.	.	** **	.	.	.	.	.	**C**	.	.	.	.	**T**	.	.	.	**T**
	**ETH2010**	**A**	**7.3**	**+**	**M133I, D144A, E164D**	**V173L, L180M, M204V**	**C**	.	.	.	.	.	.	.	.	.	.	.	.	.	.	.	.	.	.	.	.	.	** **	.	.	.	.	.	.	.	.	.	.	**T**	**A**	.	.	.
	**ETH2060**	**D**	**8.2**	**+**	**K122R, E164D**	**V173L, L180M, M204V**	.	**T**	**T**	**A**	**T**	.	.	.	.	**C**	**A**	.	.	.	.	.	.	.	.	.	.	**C**	** **	.	**G**	.	.	**C**	.	.	.	**T**	**T**	.	.	.	.	.
	ETH2110	A	3.17	-	T118A, Q129H	L82M	C	.	.	.	.	.	.	.	C	.	.	.	.	.	T	.	A	.	.	.	.	.		.	.	A	C	.	.	.	.	.	.	T	.	.	.	.
	**ETH2120**	**A**	**8.6**	**+**		**L180M, T184S, M204V,I233V**	**C**	.	.	.	.	.	.	.	.	.	.	.	.	.	.	.	.	.	.	.	.	.	** **	.	.	.	.	.	.	.	.	.	.	**T**	**A**	.	.	.
	ETH2180	D	2.74	-	G119E,T125S,C137S, T148P	V173	.	T	T	A	T	.	.	.	.	C	A	.	.	C	.	.	.	.	.	.	.	C		.	G	.	.	C	.	.	.	T	T	.	.	.	.	.
	**ETH2230**	**D**	**8.3**	**+**	**T118A, P127T, S143T**	**L80I, M204I, I233V**	.	**T**	**T**	**A**	**T**	.	.	.	.	.	**A**	.	.	.	.	.	.	.	.	.	.	**C**	** **	.	**G**	.	.	**C**	.	.	.	**T**	**T**	.	.	.	.	.
	**ETH2250**	**A**	**7.8**	**+**	**E164D**	**V173L, L180M, M204V**	**C**	.	.	.	.	.	.	.	.	.	.	.	.	.	.	.	.	.	.	.	.	.	** **	.	.	.	.	.	**C**	.	.	.	.	**T**	.	.	.	.
	**ETH2300**	**D**	**4.5**	**-**	**E164D**	**L180M, M204V**	.	**T**	.	.	.	.	.	.	.	.	.	.	.	.	.	.	.	.	.	.	.	**C**	** **	.	**G**	.	.	**C**	.	**T**	.	**T**	**T**	.	.	**A**	**A**	**T**
	**ETH2310**	**A**	**7.77**	**+**	**M103I, F134V, D144A,**	**L180M, M204V**	**C**	.	.	.	.	**G**	.	.	.	.	.	.	.	.	**T**	.	**A**	.	.	.	.	.	** **	.	.	.	.	.	**C**	.	.	.	.	**T**	.	.	.	.
	ETH2520	D	4.38	-	T118A, M133I, P127T	I233V	.	T	.	A	T	.	.	.	.	.	A	.	.	.	.	.	.	.	.	.	.	C		.	G	.	.	C	.	.	.	T	T	.	.	A	A	.
	ETH2530	D	2.64	-	T118A, S132Y	A181V, T184S	.	T	T	A	T	.	.	.	.	.	.	.	.	.	.	.	.	.	.	.	.	C		.	G	.	.	C	.	.	.	T	T	.	.	A	.	T
	**ETH2540**	**A**	**8.7**	**+**		**L180M, M204V**	**C**	.	.	.	.	.	.	.	.	.	.	.	.	.	.	.	.	.	.	.	.	.	** **	.	.	.	.	.	.	.	.	.	.	**T**	**A**	.	.	.
	**ETH3800**	**A**	**9.4**	**+**		**L180M, M204V**	**C**	.	.	.	.	.	.	.	.	.	.	.	.	.	.	.	.	.	.	.	.	.	** **	.	.	.	.	.	.	.	.	.	.	**T**	**A**	.	.	.
	**ETH3850**	**A**	**3.6**	**-**	**T143M**	**L180M, M204V**	**C**	.	.	.	.	.	.	.	.	.	.	.	.	.	.	.	.	.	.	.	.	.	** **	.	.	.	.	**C**	.	.	.	.	.	**T**	**A**	.	.	.
	**ETH3910**	**D**	**7.9**	**+**	**T127P,E164G,**	**V173L, L180M, M204V**	.	**T**	**T**	**A**	**T**	.	.	.	.	.	**A**	.	.	.	.	.	.	.	.	.	.	**C**	** **	.	.	.	.	**C**	.	.	.	**T**	**T**	.	.	.	.	.
	**ETH4340**	**D**	**4.1**	**-**	**T118A,P127T,A128V,E164D**	**L80V, V173L, L180M**	.	**T**	**T**	**A**	**T**	.	.	.	**C**	.	**A**	.	.	.	.	.	.	.	.	.	.	**C**	** **	.	**G**	.	.	**C**	.	.	.	**T**	**T**	.	.	**A**	**A**	.
	**ETH4480**	**A**	**7.7**	**+**	**Q101H,T118M, T126I,E164D**	**V173L, L180M, M204V,Q215H**	**C**	.	.	.	.	.	.	.	.	.	.	.	.	.	.	.	.	.	.	.	.	.	** **	.	.	.	.	.	.	.	.	.	.	**T**	**A**	.	.	.
	ETH4520	A	7.5	+		V191I	C	.	.	.	T	.	.	.	.	.	.	.	.	.	.	.	R	T	A	.	.	.		.	.	.	.	.	.	.	.	.	.	T	A	.	.	.
CLD patients	ETH3050	D	6.32	+	E164D	V173L	.	T	.	A	T	.	.	.	C	.	A	.	.	.	.	.	.	.	.	.	.	C		.	A	.	.	C	.	.	.	T	T	.	.	A	A	T
	ETH3380	A	4.13	-	Y100C, G130N,V168A	R138K	C	.	.	.	.	.	.	.	.	.	.	.	.	.	.	.	.	.	.	.	.	.		.	.	.	.	.	.	.	.	.	.	T	A	.	.	.
	ETH3410	D	6.8	-	L109Q, T118A, P127T	S78T,V142E	.	T	T	A	T	.	.	.	.	.	A	.	.	.	.	.	.	.	.	.	.	C		.	G	.	.	C	.	.	.	T	T	.	.	A	.	.
	ETH3500	A	4.61	+	L109I, P120T, K122R	S117Y,T128N	C	.	.	.	.	.	.	.	.	.	.	.	.	.	.	.	.	T	A	.	.	.		.	.	.	.	.	.	.	.	.	.	T	A	.	A	.
	ETH3540	D	3.89	-	P127T	I233V	.	T	T	A	T	.	.	.	.	C	A	.	.	.	T	.	A	.	.	.	.	C		.	G	.	.	C	.	.	.	T	T	.	.	A	.	.
	ETH3570	D	4.05	-	T118S	Q215P	.	T	T	A	T	.	.	.	.	C	A	.	.	.	W	.	A	.	.	.	.	C		.	G	.	.	C	.	.	.	T	T	.	.	A	.	.
	ETH3650	D	6.42	-	P120T	T128N	C	T	T	T	T	.	.	.	.	C	.	.	.	.	T	A	A	T	.	.	.	C		T	G	.	C	C	C	.	.	T	.	.	A	.	.	T
	ETH5760	D	7.34	-	T118A, P127T	I233V	.	T	T	A	T	.	.	.	.	.	A	.	.	.	T	.	A	.	.	.	.	C		.	.	.	.	C	.	.	.	T	T	.	.	R	.	.
	ETH5780	A	7.09	+	T118A, P127T	V191I, I233V	C	.	.	.	.	.	.	.	.	.	.	.	.	.	T	.	A	.	.	.	.	.		.	.	.	.	C	.	.	.	.	.	T	.	.	.	.
Blood donors	ETH2710	A	2.82	-	T127P	L82V	C	.	.	.	.	.		.	.	.	.	.	.	.	.	.	.	.	.	.	.	.		.	.	.	.	.	.	.	.	.	.	T	A	.	.	.
	ETH2720	D	2.52	-	T118A, P127T	V214A	.	T	T	A	T	.	.	.	C	.	A	.	.	.	T	.	A	.	.	.	.	C	.	.	G	.	.	C	.	.	.	.	T	.	.	A	A	T
	ETH2890	D	3.09	-	K160N, Y161F, E164G	I169L	.	T	T	A	T	G		.	.	.	A	.	.	.	.	.	.	.	.	.	.	C		.	G	.	.	C	.	.	.	T	T	.	.	A	.	.
	ETH5500	D	2.9	-	S113T,T127P	L180F	.	T	.	A	T	G	.	.	C	.	A	.	.	.	.	.	.	.	.	.	.	C	.	.	G	.	.	C	.	.	.	.	T	.	.	A	.	T
	ETH5600	A	8.44	+		V191I	C	.	.	.	.	.	.	.	.	.	.	.	.	.	T	.	A	.	.	.	.	.	.	.	.	.	.	.	.	.	.	.	.	T	A	.	.	.
	ETH1862	A	3.79	-	G130D, G130N	R138K, S219A	C	.	.	.	.	.	.	.	.	.	.	.	.	.	T	.	A	.	.	.	.	.	.	.	.	.	C	.	.	.	.	.	.	T	.	A	.	.
	ETH1871	D	3.43	+	T118A, P120T, P127T	T128N	.	T	T	A	T	.	.	.	.	.	A	.	.	.	.	.	.	.	.	C	.	.	.	.	.	.	C	.	.	.	.	.	T	.	.	A	.	.
	ETH1882	A	3.85	-	A166G	L80F	C	.	.	.	.	.	.	.	.	C	.	.	.	.	T	.	A	.	.	.	.	.		.	.	.	.	.	.	.	.	.	.	T	.	.	.	.
	ETH2042	A	3.34	-	T127P	Q215S, P237T,Q267L	C	.	.	.	.	.	.	.	.	.	.	.	.	.	.	.	.	.	.	.	.	.		.	.	.	.	.	.	.	.	.	.	T	A	.	.	.
	ETH2056	D	8.39	-	T118A, P127T, Q129H	L80F, I233V		T	T	A	T	.	.	.	.	.	A	.	.	.	.	.	A	.	.	.	.	C		.	.	.	.	C	.	.	.	T	T	.	.	A	.	.

Reference sequences of HBV genotypes of genotypes A, D and E with respective GenBank accession numbers X70185, X72702 and X75657 shown at the top of the Table to indicate the nucleotide position of interest.

*The polymerase and HBsAg escape mutant variants indicated in this Table were reported before [23].

Isolates indicated in bold were HIV co-infected individuals who developed 3TC/ telbivudine/ETV HBV drug resistance due to mutations at YMDD RT motif.

### Analysis of the BCP/PC gene variability during HIV co-infection and YMDD RT motif HBV drug resistance gene mutations

Among 48 HIV co-infected subjects analyzed, 31.3% (15) of them developed 3TC/ETV resistance at YMDD RT motif due to rtM204V/I+rtL180M and/or rtV173L HBV gene mutations (Table 4). In this HIV co-infected group, the co-prevalence of the double BCP mutant variants and the above drug resistance gene mutations was 2.1% (1/48). Similarly, the co-prevalence of YMDD RT motif 3TC/ETV resistance and the PC initiation mutant variants such as the A1814C/C1816T, G1862T and G1896A/C1858T showed 8.3% (4/48), 18.8% (9/48) and 4.2% (2/48), respectively. In addition, the co-prevalence rates of each major BCP/PC mutations and YMDD RT motif 3TC/ETV resistance gene mutations showed no significant difference when compared with the rates of BCP/PC mutations without YMDD RT motif associated 3TC/ETV resistance gene mutations, except for the overall BCP/PC mutant variants (Table 4).

**Table 4:** Frequency comparison of the BCP/PC mutant variants with and without co-prevalence of YMDD RT motif 3TC/ETV resistance mutations during HIV co-infection.

BCP/PC mutant variants	Frequency distribution, n (%)		Total,(n = 48)	P-value
	With *YMDD RT motif HBV drug resistance mutations (n = 15)	With no YMDD RT motif HBV drug resistance mutations, (n = 33)		
A1762T/G1764A	1(6.7)	3(9.1)	4(8.3)	1.00
1809–1812**	7(46.7)	21(63.6)	28(58.3)	0.35
A1814C/C1816T	4(26.7)	4(12.1)	8(16.7)	0.40
G1862T	9(60.0)	17(51.5)	26(54.2)	0.76
G1896A/C1858T	2(13.3)	7(21.2)	9(18.8)	0.70
Total	15(31.3)	33(68.8)	48(100)	<0.001

*The Kozak sequence mutants include T1809G, C1810A/T, A1811C and T1812C.

** YMDD RT motif HBV drug resistance gene mutations were reported before [23].

Other than the YMDD RT motif associated HBV drug resistance, none of the HIV co-infected patients who developed 16.7% (8/48) of ADV associated gene mutations (rtQ215H and rtI233V) revealed BCP double mutations (Table 3). In contrast, among 19 HBV mono-infected blood donors and CLD patients with drug resistance gene mutations (other than the YMDD RT motif) (Table 3), 47.4% (9/19) of them developed the BCP double mutations (Table 3). Actually, the majority of the double BCP mutations were detected from the HBV mono-infected blood donors and CLD patients who had no drug resistance gene mutations as well as genotyped as HBV A (S1 Table). In respect to PC mutations, co-distribution of the overall PC mutations and ADV resistance gene variants (rtQ215H and rtI233V) were common during HIV co-infection. However, only three HIV co-infected patients (ETH1480, ETH2120 and ETH4480) with the ADV resistance associated gene mutations developed G1862T and the other two patients (ETH1649 and ETH2520) showed mutant variant G1896A (Table 3).

Unlike HIV co-infected group, no similar comparison made among HBV mono-infected blood donors and CLD patients since none of them developed the YMDD RT motif associated 3TC/ETV resistance gene mutations (Table 3). Moreover, HBV mono-infected blood donors and CLD patients who had no any drug resistance gene variants developed comparable G1862T (60.6% (20/33) vs. 65.1% (28/43) and G1896A (24.2% (8/33) vs. 11.6% (5/43) mutant variants (S1 Table).

## Discussion

Information on the relationship between HBV BCP/PC mutations and drug resistance at YMDD RT motif and subsequent seroconversion effect during HIV co-infection and routine ART management are scarce. We reported complex patterns of HBV drug resistance and immune escape HBsAg mutant variants during HIV co-infection and ART exposure [23] using the same participants included in the current study. In the above report, 55.1% of HIV co-infected patients and 75.1% HBV mono-infected individuals were characterized by the absence of HBeAg but with detectable HBV DNA with a median viral load level of 6.39 log IU/μl and 5.63 log IU/μl, respectively. Moreover, with a proportional HBeAg positive and negative status, 29.3% of HIV co-infected patients included in the current study were reported to have 3TC/ETV resistance due to rtM204V/I+rtL180M+rtV173L mutant gene variants [23]. Therefore, the above peculiar characteristics of these HBV-HIV co-infected patients raised an interest to further characterize the co-presence of the BCP/PC genome sequence variability and HBV drug resistance gene variants at YMDD RT motifs. In addition, 43 HBV mono-infected blood donors and 52 CLD patients (with no 3TC/ETV HBV drug resistance gene mutations) were also compared for the detection of BCP/PC gene mutations.

Nevertheless, the detection of the BCP double (A1762T and G1764A) mutations was unexpectedly negligible in these HIV co-infected individuals unlike that of the HBV drug resistance gene mutations which were affected by HIV co-infection and ART exposure [23]. On the contrary, *in vitro* studies reported the evolution of the BCP double mutations was rapid during 3TC therapy from wild-type HBeAg-positive patients [6, 14] and the BCP/PC mutations generally increased HBV DNA replication levels [10, 16, 17, 25]. In view of the above facts, a similar rate of BCP double mutations was expected in these HIV co-infected patients since a high rate of HBV drug resistance mutations was reported as the result of HIV co-infection and ART exposure [23]. However, as reported before during HBV mono-infection therapy [6, 11–13], the 3TC exposure as part of ART regimens in these HIV co-infected subjects might result in reversion of the double BCP mutants to the wild-type among HBeAg-negative patients. Nevertheless, the double BCP mutants were almost absent in HBeAg positive cases, although 66.7% of the HIV co-infected patients with HBV drug resistance at YMDD RT motif were HBeAg positives. In addition, 3TC/ETV resistance at YMDD RT motif was exclusively detected in subjects with genotype A who were under ART experience [23]. Similarly, none of them showed BCP double mutations although drug resistant variants were significantly higher among subjects with genotype A irrespective of HIV co-infection status. Interestingly, this observation also contradicts to the report when patients with HBV genotype A were co-infected with HIV [26]. It could mostly be associated with BCP and PC mutations that result in a high HBeAg negativity rate and an increased HBV replication. In contrast, the common BCP mutations including the double BCP mutations were associated with the higher viral load levels and HBV genotype A among blood donors and CLD patients who showed no any drug resistance gene mutations. Moreover, mutations at nt1802 and 1803 were undetected and at nt1773 showed no significant difference in HIV co-infected patients who developed HBV 3TC resistance, despite higher BCP mutations rates were reported during 3TC therapy in HBV mono-infection [27].

In regard to PC mutations, although the overall mutant variants were found to be proportional among HIV co-infected and HBV mono-infected groups, none of the classical PC mutations (A1814C/C1816T, C1858T, G1862T and G1896) also associated with 3TC/ETV resistance gene mutations associated with the YMDD RT motif during HIV co-infection. In contrast to the current observation, *in vitro* studies [16, 27] showed higher PC mutations at the time of 3TC resistance observed as the result of 3TC therapy. Other than YMDD RT motif resistance gene mutations, the observation of co-prevalence of PC mutations and ADV resistance mutants (rtI233V, rtQ215S/Q and rtV214A) in particular in the HBeAg negative cases was in line with earlier similar reports [10, 18]. In general, the comparative analysis of the BCP/PC gene variability among 3TC/ETV resistance developed HIV co-infected patients was not in line with many of *in vitro* studies that reported 3TC therapy failure harbored the development of the BCP/PC gene mutations [10, 18]. And inversely such BCP/PC mutations can enhance viral replication efficacy of 3TC resistance-associated HBV mutations.

In contrast, both the BCP and PC mutant variants were proportionally represented in HBV mono-infected blood donors and CLD patients who experienced no any drug resistance gene mutations. Nevertheless, the principal findings in the current study can provoke a future investigation since the effects of BCP/PC mutations on viral replication might remarkably vary between different polymerase gene mutant variants. Moreover, since few *in vitro* studies [10, 18, 28, 29, 30] used specific polymerase gene mutants and independent of HBV genotypes and the combined effect of multiple gene changes as appeared in the current study subjects might have an impact on the types and patterns of BCP/PC mutations.

Regardless of HBV drug resistance and HIV co-infection, the classical BCP double mutations which were known to down-regulate transcription of PC mRNA [31] and the Kozak sequence mutants that affect HBeAg translation [32] were the predominant mutants in the current study. Moreover, unlike BCP TA4 rich (nt. 1788–1795) mutations which were absent in the current study, TA1 to TA3 rich BCP mutations [2] such as A1752C/G/T (8.4%), T1753V (18.9%), A1762T (28.7%), G1764A (35.0%) and T1773C (37.1%) were common in the current study. As the result of BCP polymorphism, clinically important multiple mutations per a study subject; such as T1753V/A1762T/G1764A and A1762T/G1764A/C1766T (or T1768A) were also the characteristics of the current study. Similarly, all sorts of PC mutant variants so far described and functionally known as the start codon mutations (A1814C/C1816; 15.4%) that abolish HBeAg expression [33] and G1862T (44.8%) mutation that interferes with post-translational modification of the HBeAg-precursor [34, 35] were most common. Interestingly, the premature stop codon (G1896A) that is known to abolish the synthesis of HBeAg [36, 37] in particular with C1858T was found to be 21.7% and commonly found in subjects with genotype D. In addition, finding a case with no multiple mutations from BCP and PC genes were difficult in the current study. However, the observation of the inverse relationship between the BCP double mutations and PC stop codon mutation was in line to a study that reported the co-existence of PC stop codon mutation might influence the BCP mutations [38].

Overall, many of the classical BCP/PC mutant variants identified in the current study were usually reported before [1–3, 5–8, 33, 38] in association with HBeAg negative CHB infection. In this regard, although the liver disease status of the HIV co-infected patients and blood donors of in the current study population were unknown, 50.0% of CLD patients were clinically defined with symptoms of liver diseases mainly ascites (11.5%), cirrhosis (7.7%), HCC (15.4%) and others (15.4%). Nevertheless, many of the BCP/PC mutations among the blood donors were comparable with CLD patients. At this moment, it was not possible to explain why for the similar percentages of HBeAg negative status and BCP/PC mutations in HBV mono-infected blood donors and CLD patients since the former study participants were not assessed for their liver disease status. This could be taken as the limitation of the current study. Irrespective of the study groups, the majority of the classical BCP/PC mutants such as the Kozak sequences mutant, PC initiation and G1896A with C1858T mutations showed significantly higher rate in HBeAg negative subjects. However, in most cases, higher viral load levels and HBeAg positivity showed a significant correlation to the major BCP/PC mutations. These could also be an indication of a wild-type mixed HBV viral population infection, but with BCP/PC mutant variants [39]. In addition, the development of such mutant variants in both HBeAg positive and negative case could be the result of the synergistic effect of different mutation profiles or genotype differences [37, 40]. In the current study, such mutant profiles were particularly seen in association with genotype D except for the double BCP mutations.

In conclusion, the higher record of BCP/PC gene variability irrespective of HIV co-infection and HBV drug resistance indicates a high risk of potential HBeAg negative chronic HBV infection in this setting. Except for the double BCP mutation which was significantly lower in HIV co-infected patients, the Kozak sequences BCP mutants and the majority of PC mutations showed no significant differences among the study groups. Moreover, the co-prevalence rates of each major BCP/PC mutations and YMDD RT motif associated 3TC/ETV resistance gene mutations showed no significant differences when compared with the rates of BCP/PC mutations without YMDD RT motif 3TC/ETV resistance genes during HIV co-infection. Therefore, the current study further confirmed a more impact and influence of HIV co-infection and unintended ART exposures on HBV drug resistance genes than the BCP/PC genes variability in this setting. However, whether such observation is due to an absence of a true correlation or a combined effect of genotypes and a presence of complex mutant variants, a further longitudinal prospective study is needed with a little bit larger sample size.

## Supporting information

S1 Table Comparison of HBV BCP and PC nucleotide sequences changes with respect to HIV co-infected patients (n = 20), liver disease patients (n = 43) and blood donors (n = 33) with no HBV drug resistance polymerase gene mutations. (DOC)

S1 Fig A phylogenetic tree of BCP/PC sequences covering 307 nucleotides length (nt 1653–1959) of the partial X protein, full precore and partial core protein (C) of HBV genome regions. The analysis involved 184 nucleotide sequences; 143 from study sequences (identified as ETH followed by four digit numbers) and 40 reference sequences representing respective HBV genotypes. The reference sequences are designated by their respective accession numbers and country of origin along HBV genotype. The genome of the Woolly monkey HBV (GenBank AY226578; marked) was utilized as an out-group. Bootstrap statistical analysis was performed from 1000 replicates, indicated as percentages on the nodes (Bootstrap values < 70% were not shown in the tree). The phylogenetic analysis was conducted in MEGA6 (http://www.megasoftware.net/) using the Neighbor-Joining method.
(TIF)
