# Supplementary material for: Analysis of HBV basal core promoter/precore gene variability in patients with HBV drug resistance and HIV co-infection in Northwest Ethiopia
Source: PLoS One. 2018 Feb 6;13(2):e0191970. doi: 10.1371/journal.pone.0191970 (PMC5800642; doi:10.1371/journal.pone.0191970)
Supplement: S1 Table — (DOC) [file pone.0191970.s001.doc]

|  |  | | | | |  | BCP transcriptional & translational genes | | | | | | | | | | | | | | | | | | | | | | | | | | PC initation & post translational genes | | | | | | | |  |
| --- | --- | --- | --- | --- | --- | --- | --- | --- | --- | --- | --- | --- | --- | --- | --- | --- | --- | --- | --- | --- | --- | --- | --- | --- | --- | --- | --- | --- | --- | --- | --- | --- | --- | --- | --- | --- | --- | --- | --- | --- | --- |
|  |  |  |  |  |  | 1 | 1 | 1 | 1 | 1 | 1 | 1 | 1 | 1 | **1** | 1 | 1 | 1 | **1** | 1 | **1** | 1 | 1 | 1 | 1 | 1 | 1 | 1 | 1 | 1 | 1 | 1 | **1** | 1 | 1 | 1 | **1** | 1 | 1 | **1** | 1 |
| Study groups |  |  |  |  |  | 6 | 6 | 6 | 7 | 7 | 7 | 7 | 7 | 7 | 7 | 7 | 7 | 7 | 7 | 7 | 7 | 7 | 7 | 7 | 7 | 7 | 7 | 7 | 8 | 8 | 8 | 8 | 8 | 8 | 8 | 8 | 8 | 8 | 8 | 8 | 8 |
|  |  |  |  |  |  | 7 | 7 | 7 | 0 | 1 | 2 | 3 | 4 | 5 | 5 | 5 | 6 | 6 | 6 | 6 | 6 | 6 | 6 | 7 | 7 | 7 | 7 | 9 | 0 | 1 | 1 | 1 | 1 | 1 | 4 | 5 | 5 | 6 | 8 | 9 | 9 |
|  | Isolate | Genotype | VL | HBeAg | HBsAg escape mutations* | 4 | 6 | 8 | 3 | 9 | 7 | 0 | 1 | 2 | 3 | 7 | 0 | 1 | 2 | 3 | 4 | 6 | 8 | 1 | 2 | 3 | 5 | 9 | 9 | 0 | 1 | 2 | 4 | 6 | 5 | 0 | 8 | 2 | 8 | 6 | 9 |
| Ref.strians | X70185 | A |  |  |  | T | A | C | C | G | G | C | T | A | T | G | A | A | A | G | G | C | T | T | A | T |  | C | G | C | A | C | A | G | C | A | C | G | G | G | G |
|  | X72702 | D |  |  |  | T | . | . | A | T | . | . | . | . | . | A | . | . | . | . | . | . | . | . | . | C |  | . | . | . | . | . | . | . | . | T | T | . | . | . | . |
|  | X75657 | E |  |  |  | T | T | . | A | T | A | . | C | . | . | A | . | . | . | . | . | . | . | . | . | C |  | . | . | . | . | . | . | . | . | T | T | . | . | . | . |
| HBV-HIV coinfected | ETH1160 | A | 8.49 | - |  | . | . | . | . | . | . | . | . | . | . | . | . | . | . | . | . | . | . | . | . | . |  | . | . | . | . | . | . | . | . | . | . | T | A | . | . |
|  | ETH1560 | A | 7.57 | + | E164G | . | . | . | . | . | . | . | . | . | . | . | . | . | . | . | . | . | . | . | . | . |  | . | . | . | . | . | . | . | . | . | . | T | A | . | . |
|  | ETH1570 | A | 8.93 | - | D144E, G145R | . | . | . | . | . | . | . | . | . | . | . | . | . | . | . | . | . | . | . | . | . |  | . | . | . | . | . | C | . | . | . | . | T | . | . | . |
|  | ETH1580 | D | 3.98 | - | T118A, P127T | T | T | T | A | T | . | . | . | . | C | A | . | . | . | . | . | . | . | . | . | C |  | . | G | . | . | C | . | . | . | T | T | . | . | . | . |
|  | ETH1700 | A | 2.72 | - | F158FS, A166G | . | . | . | . | . | . | . | . | . | . | . | . | . | . | . | . | . | . | . | . | . |  | . | . | . | . | . | . | . | . | . | T | T | . | A | A |
|  | ETH1890 | D | 3.07 | - |  | T | T | T | A | T | . | . | . | . | . | A | . | . | . | . | . | . | . | . | . | C |  | . | G | . | . | C | . | . | . | T | T | . | . | . | . |
|  | ETH2091 | A | 8.41 | + | T131I | . | . | . | . | . | . | . | . | . | . | . | . | . | . | . | . | . | . | . | . | . |  | . | . | . | . | . | . | . | . | . | . | T | A | . | . |
|  | ETH2130 | D | 8.84 | - | Q101R, T118A,T127A, Q129R G159A,R169H | T | T | T | A | T | . | . | . | M | K | A | . | . | . | . | . | . | . | . | . | C |  | . | G | . | . | C | . | . | . | T | T | . | . | A | . |
|  | ETH2190 | A | 2. 57 | - |  | . | . | . | . | . | . | . | . | . | C | . | . | . | T | . | A | . | . | . | . | . |  | . | . | . | C | . | . | . | . | T | . | T | A | . | . |
|  | ETH2220 | A | 2.98 | + | L109P | . | . | . | . | . | . | . | . | . | . | . | . | . | . | . | . | T | A | . | . | . |  | . | . | . | . | . | . | . | . | . | . | T | A | . | . |
|  | ETH2610 | A | 8.91 | + |  | . | . | . | . | . | . | . | . | . | . | . | . | . | . | . | . | . | . | . | . | . |  | . | . | . | . | . | . | . | . | . | . | T | A | . | . |
|  | ETH3590 | A | 5,11 | - | Y161F, Q181E | . | . | . | . | . | . | . | . | . | . | . | . | . | . | . | A | T | A | . | . | . |  | . | . | . | . | . | . | . | . | . | . | T | A | . | . |
|  | ETH3660 | D | 3,85 | - | T118A, P127T | T | T | T | A | . | . | . |  |  |  | A | . | . | . | . | . | . | . | . | . | C |  |  | G | . | . | . | . | . | . | T | T |  |  | . | . |
|  | ETH3720 | A | 8,4 | + |  | . | . | . | . | . | G | . | . | . | . | . | C | . | . | . | . | . | . | . | . | . |  | . | . | . | M | . | . | . | . | . | . | T | A | . | . |
|  | ETH3750 | A | 2,94 | - | T127P | . | . | . | . | . | . | . | . | . | . | . | . | . | . | . | . | . | . | . | . | . |  | . | . | . | . | . | C | . | . | . | . | T | A | . | A |
|  | ETH3790 | A | 3,62 | - |  | . | . | . | . | . | . | . | . | . | . | . | . | . | . | . | . | . | . | . | . | . |  | . | . | . | . | . | C | . | . | . | . | T | . | . | . |
|  | ETH3840 | A | 4,38 | - | T114K, M133T | . | . | . | . | . | . | . | . | . | . | . | . | . | . | . | . | . | . | . | . | . |  | . | . | . | . | . | . | . | . | . | . | T | A | . | . |
|  | ETH3870 | A | 8,67 | - | L109V | . | . | . | . | . | G | . | . | . | . | . | . | . | T | . | A | T | . | . | . | . |  | . | . | . | C | . | . | . | . | T | . | T | . | . | A |
|  | ETH4500 | D | 3,08 | - | T127P | **T** | **T** | **T** | **A** |  |  | **.** |  |  |  | **A** |  |  |  |  |  |  |  |  |  | **C** |  |  | **G** |  |  | **C** |  |  |  | **T** | **T** |  |  |  |  |
|  | ETH4530 | D | 8,73 | - | T127P, T189I, F220C | T | T | . | A | T | G | . | . | C | . | A | . | . | . | . | . | . | . | . | C | C |  | . | G | . | . | C | . | . | . | T | T | . | . | A | A |
| CLD patients | ETH3070 | A | 2,58 | - | T127P,T189I | . | . | . | . | . | . | . |  |  | . | . | . | . | . | . | . | . | . | . | . | . | . |  | . | . | . | C | . | . | . | T | . | . | . | . | . |
|  | ETH3090 | A | 4,08 | - | T127P, Y134F, S210R | . | . | . | . | . | . | . | . | . | . | . | . | . | . | . | . | T | A | . | . | . |  | . | . | . | . | . | . | . | . | . | . | T | A | . | . |
|  | ETH3110 | A | 7,44 | + | Q129HR, G130N | . | . | . | . | . | . | . | . | . | . | . | . | . | T | . | A | . | . | . | . | . |  | . | G | T | . | . | . | . | . | . | . | T | C | . | . |
|  | ETH3120 | A | 6,81 | + | M133T,T140I | . | . | . | . | . | . | . | . | . | G | . | . | . | . | . | A | T | A | . | . | . |  | . | . | A | . | . | . | . | T | C | A | T | T | . | C |
|  | ETH3130 | D | 6,26 | - | T118A, P127T | T | T | T | A | T | . | . | . | . | C | A | . | . | T | . | A | . | . | . | . | C |  | . | G | . | . | C | . | . | . | T | T | . | . | A | A |
|  | ETH3170 | D | 8,52 | - | T118A, P127T | T | T | T | A | A | . | . | . | . | . | A | . | . | . | . | . | . | . | . | . | C | . | . | G | . | . | C | . | . | . | T | . | . | . | A | . |
|  | ETH3250 | A | 2,85 | + |  | . | . | . | . | . | . | . | . | . | . | . | . | . | . | . | . | . | . | . | . | . |  | . | . | . | . | . | C | . | . | T | . | T | . | . | A |
|  | ETH3290 | D | 6,12 | + | T118A, P127T | T | T | T | A | T | . | . | . | . | . | A | . | . | . | . | . | . | . | . | . | C |  | . | G | . | . | C | . | . | . | T | T | . | . | . | . |
|  | ETH3300 | A | 7,19 | - |  | . | . | . | . | . | . | . | . | . | . | . | . | . | T | . | A | . | . | . | . | . | . | . | . | . | . | . | . | . | . | . | . | T | A | . | . |
|  | ETH3310 | A | 2,98 | - |  | . | . | . | . | . | . | . | . | . | . | . | . | . | T | . | A | . | . | . | . | . | . | . | . | . | . | . | . | . | . | . | . | T | A | . | . |
|  | ETH3340 | D | 3,95 | - | T118A, P127T | T | T | T | A | T | . | . | . | . | . | A | . | . | . | . | . | . | . | . | . | C | . | . | . | . | . | C | . | . | . | T | T | . | . | . | . |
|  | ETH3370 | D | 3,04 | - | T118A, P127T | T | T | T | A | T | . | . | . | M | . | A | . | . | . | . | . | . | . | . | . | C |  | . | G | . | . | C | . | . | . | T | T | . | . | R | R |
|  | ETH3430 | D | 2,35 | - | T118A, T126S, P127T | T | T | T | . | T | . | . | . | . | C | . | . | . | T | A | A | Y | . | . | . | C |  | . | G | . | . | C | . | . | . | T | T | . | . | R | A |
|  | ETH3431 | D | 2,35 | - | T127P, Y134N | T | T | T | A | T | . | . | . | . | . | A | . | . | . | . | . | . | . | . | . | C | . | . | G | . | . | . | . | . | . | . | T | . | . | . | . |
|  | ETH3450 | A | 6,97 | + | T127P,Y134N | . | . | . | . | . | . | . | . | . | . | . | . | . | . | . | . | . | . | . | . | . |  | . | . | . | . | . | . | . | . | . | . | T | A | . | . |
|  | ETH3480 | A | 5,46 | + | Y161F | . | . | . | A | . | . | . | . | . | . | . | . | . | T | . | A | Y | W | . | . | . |  | . | . | . | . | . | . | . | . | . | . | T | . | . | . |
|  | ETH3530 | A | 6,89 | - | Y161F | . | . | . | A | . | . | . | . | . | . | . | . | . | T | . | A | . | . | . | . | . |  | . | . | . | . | . | . | . | . | . | . | T | . | . | . |
|  | ETH3550 | A | 3 | - | Y161F | . | . | . | A | . | . | . | . | . | . | . | . | . | T | . | A | Y | . | . | . | . |  | . | . | . | . | . | . | . | . | . | . | T | . | . | . |
|  | ETH3560 | A | 6,01 | + |  | . | . | . | . | . | . | . | . | . | . | . | . | . | . | . | A | T | A | . | . | . |  | . | . | . | . | . | . | . | . | . | . | T | A | . | . |
|  | ETH3580 | A | 5,47 | + | P127T, F134Y,Y161F | . | . | . | . | . | . | . | . | . | . | . | . | . | . | . | A | T | A | . | . | . |  | . | . | . | . | . | . | . | . | . | . | T | A | . | . |
|  | ETH3600 | A | 6,66 | + | Y161F | . | . | . | A | . | . | . | . | . | . | . | . | . | T | . | A | . | . | . | . | . | . | . | . | . | . | C | . | . | . | . | . | T | . | . | . |
|  | ETH3610 | A | 7,22 | - |  | . | . | . | . | . | . | . | . | . | . | . | . | . | . | . | . | . | . | . | . | . |  | . | . | . | C | . | . | . | . | . | . | T | C | . | . |
|  | ETH3640 | A | 4,03 | - | Y161F | . | . | . | . | . | . | . | . | . | . | . | . | . | . | . | . | . | . | . | . | . |  | . | . | . | . | . | C | . | . | . | . | T | . | . | . |
|  | ETH3700 | A | 7,6 | + |  | . | . | . | . | . | . | T | . | . | . | . | . | . | T | . | . | T | . | . | . | . |  | . | . | . | . | . | . | . | . | . | . | T | A | . | . |
|  | ETH4040 | A | 4,18 | - |  | . | . | . | . | . | . | . | . | . | . | . | . | . | . | . | . | T | . | . | . | . |  | . | . | . | . | . | . | . | . | . | T | T | . | . | A |
|  | ETH4060 | A | 6,34 | - | Y161F | . | . | . | . | . | . | . | . | . | . | . | . | . | T | . | . | T | A | . | . | . |  | . | . | . | . | . | . | . | . | . | . | T | A | . | . |
|  | ETH4070 | D | 4,04 | - | P127T | T | . | . | A | T | . | . | . | . | C | A | . | . | . | . | R | . | . | . | . | . |  | . | K | . | . | Y | . | . | . | T | Y | K | . | A | . |
|  | ETH4080 | D | 2,89 | - | T118A, P127T | T | T | T | A | T | . | . | . | . | . | A | . | . | . | . | . | . | . | . | . | C | . | . | G | . | . | C | . | . | . | . | T | . | . | . | . |
|  | ETH4100 | A | 3,15 | - |  | . | . | . | . | . | . | . | . | . | . | . | . | . | . | . | . | . | . | . | . | . | . | . | . | . | . | . | . | . | . | . | T | T | A | A | . |
|  | ETH4130 | D | 4,43 | - |  | T | T | T | A | T | . | . | . | . | . | A | . | . | . | . | . | . | . | . | . | C | . | . | G | . | . | C | . | . | . | . | T | . | . | . | . |
|  | ETH4140 | A | 4,3 | + |  | . | . | . | . | . | . |  | . | . | . | . | . | . | . | . | . | . | . | . | . | . |  | . | . | . | . | . | . | . | . | . | . | T | A | . | . |
|  | ETH5670 | A | 8,26 | + |  | . | . | . | . | . | . | . | . | . | . | . | . | . | . | . | . | . | . | . | . | . | . | . | . | . | . | . | . | . | . | . | . | T | A | . | . |
|  | ETH5700 | A | 5,87 | + |  | . | . | . | . | . | . | . | . | . | . | . | . | . | . | . | A | . | . | . | . | . | . | . | . | . | . | . | . | . | . | . | . | T | A | . | . |
|  | S1 Tabble (continued) | | | | HBsAg escape gene mutations* |  | | | | | | | | | | | | | | | | | | | | | | | | | | |  | | | | | | | | |
|  |  | | | |  | BCP transcriptional & translational genes | | | | | | | | | | | | | | | | | | | | | | | | | | | PC initation & post translational genes | | | | | | | | |
|  |  |  |  |  |  | 1 | 1 | 1 | 1 | 1 | 1 | 1 | 1 | 1 | **1** | 1 | 1 | 1 | **1** | 1 | **1** | 1 | 1 | 1 | 1 | 1 | 1 | 1 | 1 | 1 | 1 | 1 | **1** | 1 | 1 | 1 | **1** | 1 | 1 | **1** | 1 |
|  |  |  |  |  |  | 6 | 6 | 6 | 7 | 7 | 7 | 7 | 7 | 7 | 7 | 7 | 7 | 7 | 7 | 7 | 7 | 7 | 7 | 7 | 7 | 7 | 7 | 7 | 8 | 8 | 8 | 8 | 8 | 8 | 8 | 8 | 8 | 8 | 8 | 8 | 8 |
|  |  |  |  |  |  | 7 | 7 | 7 | 0 | 1 | 2 | 3 | 4 | 5 | 5 | 5 | 6 | 6 | 6 | 6 | 6 | 6 | 6 | 7 | 7 | 7 | 7 | 9 | 0 | 1 | 1 | 1 | 1 | 1 | 4 | 5 | 5 | 6 | 8 | 9 | 9 |
|  | Isolates | Genotype | VL | HBeAg |  | 4 | 6 | 8 | 3 | 9 | 7 | 0 | 1 | 2 | 3 | 7 | 0 | 1 | 2 | 3 | 4 | 6 | 8 | 1 | 2 | 3 | 5 | 9 | 9 | 0 | 1 | 2 | 4 | 6 | 5 | 0 | 8 | 2 | 8 | 6 | 9 |
|  | X70185 | A |  |  |  | T | A | C | C | G | G | C | T | A | T | G | A | A | A | G | G | C | T | T | A | T |  | C | G | C | A | C | A | G | C | A | C | G | G | G | G |
|  | X72702 | D |  |  |  | T | . | . | A | T | . | . | . | . | . | A | . | . | . | . | . | . | . | . | . | C |  | . | . | . | . | . | . | . | . | T | T | . | . | . | . |
|  | X75657 | E |  |  |  | T | T | . | A | T | A | . | C | . | . | A | . | . | . | . | . | . | . | . | . | C |  | . | . | . | . | . | . | . | . | T | T | . | . | . | . |
|  | ETH5730 | D | 4,47 | - | T118A, P127T | . | C | . | A | . | . | . | . | . | . | R | . | . | . | . | . | . | . | . | . | . |  | . | . | . | . | C | . | . | . | Y | T | . | . | . | . |
|  | ETH5740 | D | 5,68 | + | T118A, P127T,T189I | T | T | T | A | T | . | . | . | . | . | R | . | . | T | . | A | . | A | . | . | C |  | . | G | . | . | C | . | . | . | T | T | . | . | . | . |
|  | ETH5770 | A | 6,02 | + | V106G | . | . | . | . | . | . | . | . | . | . | . | . | . | T | . | A | . | . | . | . | . |  | . | . | . | . | G | . | . | . | . | . | T | A | . | . |
|  | ETH5790 | A | 5,01 | - |  | . | . | . | . | . | . | . | . | . | . | . | . | . | . | . | . | . | . | . | . | . |  | . | . | . | . | . | C | . | . | . | . | T | T | . | . |
|  | ETH5820 | A | 4,63 | + |  | . | . | . | . | . | . | . | . | . | . | . | . | . | . | . | . | . | . | . | . | . |  | . | . | . | . | . | . | . | . | . | . | T | A | . | . |
|  | ETH5830 | A | 3,54 | - |  | . | . | . | . | . | . | . | . | . | C | . | . | . | T | . | A | . | . | . | . | . |  | . | . | . | C | . | . | . | . | . | . | T | . | . | R |
|  | ETH5840 | A | 4,34 | + | S210R | . | . | . | . | . | . | . | . | . | C | . | . | . | . | . | A | T | A | . | . | . |  | . | . | . | T | . | . | . | . | . | . | T | A | . | . |
|  | ETH5860 | A | 6,5 | + | T118A | . | . | . | . | . | . | . | . | . | . | . | . | . | T | . | A | Y | . | . | . | . |  | . | . | . | . | C | . | . | . | . | . | T | . | . | . |
|  | ETH6010 | D | 6,93 | - | T127P | T | T | . | A | T | G | . | . | . | C | A | . | . | . | . | . | . | . | . | . | C |  | . | G | . | . | C | . | . | . | T | T | . | . | A | A |
|  | ETH6020 | D | 2,58 | - | T114P | T | T | T | A | T | . | . | . | . | . | A | . | . | . | . | . | . | . | . | . | C | . | . | K | . | . | C | . | . | . | T | T | . | . | . | . |
|  | ETH2380 | D | 3,1 | - | T114P | T | T | T | A | T |  | . |  |  |  | A |  |  |  |  |  |  |  |  |  | C |  |  |  |  |  | C |  | T |  | T | T |  |  | A |  |
| Blood donors | ETH2480 | A | 3,79 | - | S113T | . | . | . | . | . | . | . | . | . | . | . | . | . | T | . | A | . | . | . | . | . | . | . | . | . | . | . | . | . | . | . | . | T | A | . | . |
|  | ETH2510 | A | 2,92 | - | A159V | . | . | . | . | . | . | . | . | . | . | . | . | . | T | . | A | . | . | . | . | . | . | . | . | . | . | . | . | . | . | . | . | T | . | . | . |
|  | ETH2670 | D | 3,31 | - | T118A, P127T | T | T | T | A | T | . | . | . | . | . | R | . | . | T | . | A | . | . | . | . | C |  | . | . | . | . | C | . | . | . | T | T | . | . | A | A |
|  | ETH2680 | A | 3,4 | - | P127T | . | . | . | . | . | . | . | . | . | . | . | . | . | . | . | A | T | . | . | . | . | . | . | . | . | . | C | . | . | . | . | . | T | A | . | . |
|  | ETH2740 | A | 2,96 | - |  | . | . | . | . | . | . | . | . | . | . | . | . | . | . | . | . | . | . | . | . | . | . | . | . | T | C | . | . | . | . | T | . | T | . | . | . |
|  | ETH2750 | A | 3,18 | - | T118A, P127T, L216* | . | . | . | . | . | . | . | . | . | . | . | . | . | . | . | . | . | . | . | . | . |  | . | . | . | . | . | C | . | . | . | . | T | . | . | . |
|  | ETH2770 | D | 3,36 | - | T118A, P127T, A159V | T | T | T | A | T | . | . | . | . | . | A | . | . | . | . | . | . | . | . | . | C | . | . | G | . | . | C | . | . | . | T | T | . | . | . | A |
|  | ETH2780 | D | 3,79 | - | T118A, P127T, G159A | T | T | T | A | T | . | . | . | . | C | . | . | M | . | . | R | . | . | . | . | C |  | . | G | . | . | C | C | . | . | T | T | . | . | A | A |
|  | ETH2800 | A | 2,8 | - |  | . | . | . | . | . | . | . | . | . | A | . | . | . | T | . | . | T | A | . | . | . |  | . | . | T | C | . | . | . | . | . | . | T | A | . | . |
|  | ETH2830 | A | 7,09 | - | E164G,T189I | . | . | . | . | . | . | . | . | . | . | . | . | . | T | . | A | . | . | . | . | . |  | . | . | . | . | . | . | . | . | . | . | T | . | . | . |
|  | ETH2850 | D | 2,7 | + | T118A, P127T | T | T | T | A | T | G | . | . | . | . | A | R | . | . | . | . | . | . | . | . | C | R | . | G | . | . | C | . | . | . | T | T | . | . | . | . |
|  | ETH2860 | D | 3,76 | - | T118A, P127T | T | T | T | A | T | . | . | . | . | . | A | . | . | . | . | . | . | . | . | . | C |  | . | G | . | . | C | . | . | . | T | T | . | . | A | . |
|  | ETH2870 | D | 3,14 | - | T127P | T | T | T | A | . | G | . | . | . | . | A | . | . | . | . | A | . | . | . | . | C | . | . | G | . | . | C | . | . | . | T | T | . | . | . | A |
|  | ETH2880 | A | 3,7 | - |  | T | . | . | . | . | . | . | . | . | . | . | . | . | . | . | . | . | . | . | . | . | . | . | G | . | . | C | . | . | . | T | . | . | . | . | A |
|  | ETH2910 | A | 2,77 | + |  | . | . | . | . | . | G |  | . | . | . | . | . | . | . | . | A | T | . | . | . | . |  | . | . | T | C | . | . | . | . | . | . | T | A | . | . |
|  | ETH2930 | D | 3,35 | - |  | T | T | T | A | T | . | A | . | C | . | A | . | . | . | . | . | . | . | . | . | C | . | . | G | . | . | C | . | . | . | T | T | . | . | A | . |
|  | ETH2960 | A | 2,72 | - |  | . | . | . | . | . | . | . | . | . | . | . | . | . | . | . | . | . | . | . | . | . | . | . | . | . | . | . | C | . | . | . | . | T | A | . | . |
|  | ETH2980 | A | 3,57 | - | D144A | T | T | T | A | T | . | . | . | . | . | A | . | . | . | . | . | . | . | . | . | C |  | . | G | . | . | C | . | . | . | T | T | . | . | A | . |
|  | ETH3690 | A | 5,29 | + | T189I | . | . | . | A | . | . |  | . | . | . | . | . | . | T | . | A | . | . | . | . | . |  | . | . | . | . | Y | . | . | . | . | . | T | A | . | . |
|  | ETH5460 | A | 3,25 | + |  | . | . | . | . | . | . | . | . | . | . | . | . | . | T | . | A | . | . | . | . | . | . | . | . | . | C | . | . | . |  | T | . | T | . | . | . |
|  | ETH5520 | A | 8,61 | + |  | . | . | . | . | . | . | . | . | . | . | . | . | . | T | . | A | . | . | . | . | . | . | . | . | . | . | . | . | . | . | . | . | T | A | . | . |
|  | ETH5540 | A | 2,82 | - |  | . | . | . | . | . | . | . | . | G | . | T | . | . | T | A | . | T | . | C | . | . |  | . | . | . | . | . | . | . | . | . | . | T | A | . | . |
|  | ETH5550 | A | 2,9 | - | Q101H, N13,Y161F | . | . | . | . | . | . | . | . | . | G | . | . | . | T | . | A | . | . | . | . | . |  | . | . | . | C | . | . | . | . | . | . | T | . | . | . |
|  | ETH5580 | A | 8,84 | - | T118P | . | . | . | . | . | . | . | . | . | . | . | . | . | . | . | . | . | . | . | . | . |  | . | . | . | . | . | . | . | . | . | . | T | A | . | . |
|  | ETH1991 | A | 3,78 | + | T127P | **.** | **.** | **.** | **.** | **.** | **.** | **.** | **.** | **.** | **.** | **.** | **.** | **.** | **T** | **.** | **A** | **.** | **.** | **.** | **.** | **.** | **.** | **.** | **.** | **.** | **.** | **.** | **.** | **.** | **.** | **.** | **.** | **T** | **A** | **.** | **.** |
|  | ETH1994 | A | 3,9 | + |  | . | . | . | . | . | . | . | . | . | . | . | . | . | T | . | A | . | . | . | . | . |  | . | . | . | . | . | . | . | . | . | . | T | A | . | . |
|  | ETH2048 | D | 3,46 | - |  | . | . | . | A | T | . | . | . | . | . | R | . | . | . | . | . | . | . | . | . | Y |  | . | Y | . | . | Y | . | . | . | W | Y | . | R | R | R |
|  | ETH2059 | A | 3,24 | - | L216* | . | . | . | . | . | . | . | . | . | . | . | . | . | . | . | . | . | . | . | . | . |  | . | . | . | . | . | . | . | . | . | . | T | A | . | . |
|  | ETH2090 | A | 9,01 | + |  | . | . | . | . | . | . | . | . | . | . | . | . | . | . | . | . | . | . | . | . | . |  | . | . | . | . | . | . | . | . | . | . | T | A | . | . |
|  | ETH2121 | D | 2,78 | - | T118A, P127T, A166P | T | T | T | A | T | . | . | . | . | . | R | . | . | . | . | . | . | . | . | . | C |  | . | G | . | . | C | . | . | . | T | T | . | . | A | . |
|  | ETH2138 | D | 8,96 | + | T127P | . | . | . | A | T | . | . | . | . | . | . | . | . | . | . | . | . | . | . | . | . | . | . | K | . | . | C | . | K | . | W | Y | K | . | A | . |
|  | ETH2283 | A | 4,88 | - |  | . | . | . | . | . | . | . | . | . | . | A | . | . | T | . | A | . | . | . | . | . | . | . | . | . | . | . | . | . | . | . | . | T | A | . | . |

Reference sequences of HBV genotypes of genotypes A, D and E with respective GenBank accession numbers X70185, X72702 and X75657 shown at the top of the Table to indicate the nucleotide position of interest.

*The HBsAg escape gene mutant variants indicated in this Table were reported before [23].
