# Supplementary figures and images for: Analysis of HBV basal core promoter/precore gene variability in patients with HBV drug resistance and HIV co-infection in Northwest Ethiopia
Source: PLoS One. 2018 Feb 6;13(2):e0191970. doi: 10.1371/journal.pone.0191970 (PMC5800642; doi:10.1371/journal.pone.0191970)

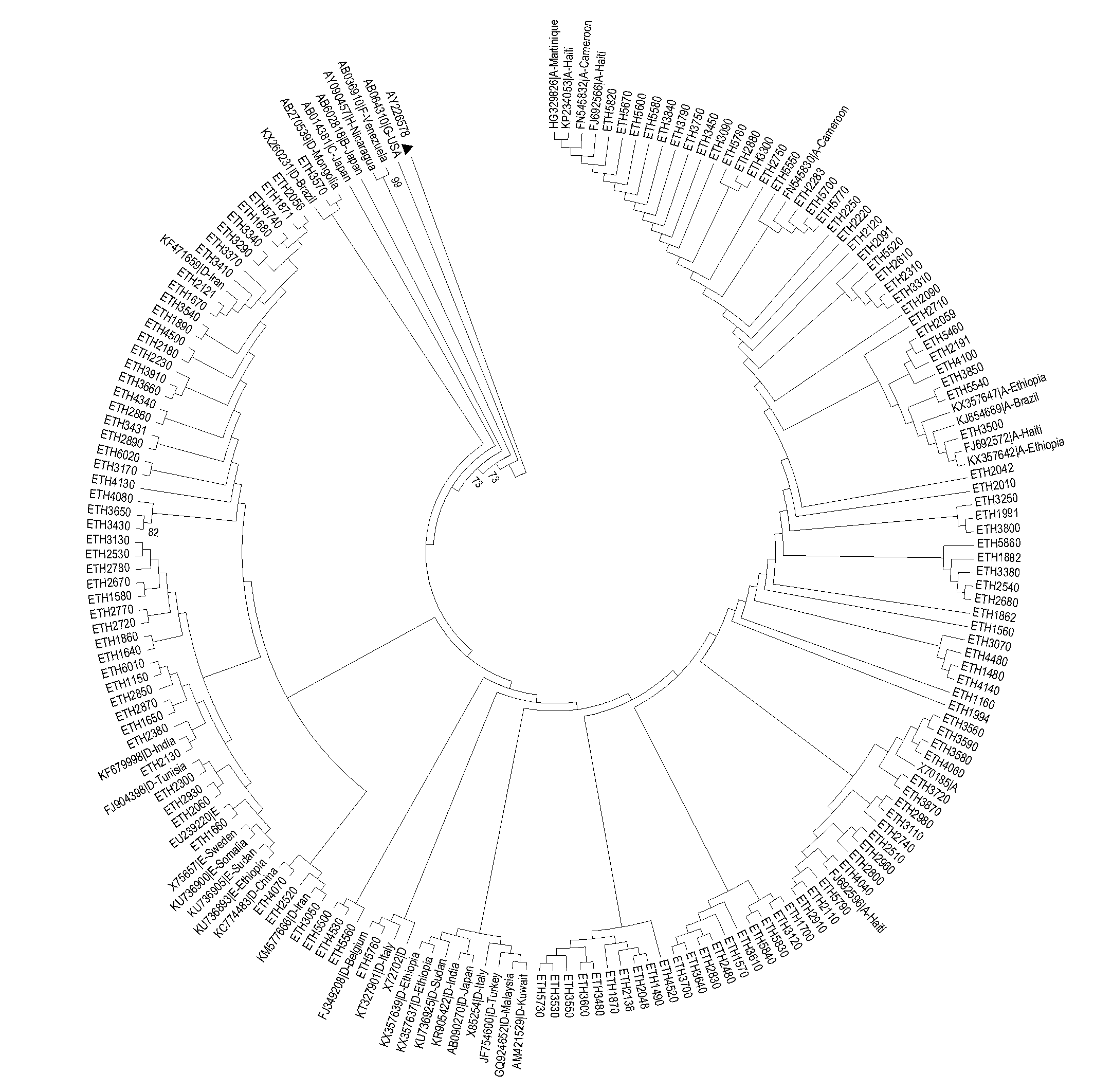

Supplement: S1 Fig — The analysis involved 184 nucleotide sequences; 143 from study sequences (identified as ETH followed by four digit numbers) and 40 reference sequences representing respective HBV genotypes. The reference sequences are designated by their respective accession numbers and country of origin along HBV genotype. The genome of the Woolly monkey HBV (GenBank AY226578; marked) was utilized as an out-group. Bootstrap statistical analysis was performed from 1000 replicates, indicated as percentages on the nodes (Bootstrap values < 70% were not shown in the tree). The phylogenetic analysis was conducted in MEGA6 (http://www.megasoftware.net/) using the Neighbor-Joining method. (TIF) [file pone.0191970.s002.tif]
